# Oncologic and surgical outcomes in colorectal cancer patients with liver cirrhosis: A propensity-matched study

**DOI:** 10.1371/journal.pone.0178920

**Published:** 2017-06-06

**Authors:** Eon Chul Han, Seung-Bum Ryoo, Ji Won Park, Jin Wook Yi, Heung-Kwon Oh, Eun Kyung Choe, Heon-Kyun Ha, Byung Kwan Park, Sang Hui Moon, Seung-Yong Jeong, Kyu Joo Park

**Affiliations:** 1 Department of Surgery, Dongnam Institute of Radiological and Medical Sciences, Busan, Korea; 2 Department of Surgery, Seoul National University College of Medicine, Seoul, Korea; 3 Department of Surgery, Seoul National University Bundang Hospital, Seoul National University College of Medicine, Seongnam, Korea; 4 Seoul National University Hospital Gangnam Center, Seoul, Korea; 5 Department of Surgery, Seonam University College of Medicine Myongji Hospital, Goyang, Gyeonggi Province, Korea; 6 Department of Surgery, Chung-Ang University Hospital, Seoul, Korea; Chang Gung Memorial Hospital Kaohsiung Branch, TAIWAN

## Abstract

The management of colorectal cancer in patients with liver cirrhosis requires a thorough understanding of both diseases. This study evaluated the effect of liver cirrhosis on oncologic and surgical outcomes and prognostic factors in colorectal cancer patients. Fifty-five consecutive colorectal cancer patients with liver cirrhosis underwent colorectal resection (LC group). Using a prospectively maintained database, these patients were matched 1:4 using propensity scoring with R programming language, package "MatchIt" and "optmatch" by sex, age, cancer location, and tumor stage with 220 patients without liver cirrhosis (non-LC group), resulting in 275 patients. The 5-year overall survival (OS) was significantly worse in the LC group than in the non-LC group (46.7% vs. 76.2% respectively, P < 0.001); however, the 5-year proportion of recurrence free (PRF) rates were similar (73.1% vs. 84.5% respectively, P = 0.094). On multivariate analysis of the LC group, tumor-node-metastasis (TNM) stage ≥III disease, venous invasion, and a model for end-stage liver disease plus serum sodium (MELD-Na) score >10 were prognostic factors for OS. However, the OS was not different between the LC group with MELD-Na score ≤10 and the non-LC group (5-year OS rate, TNM stage ≤II, 85.7 vs 89.5%, p = 0.356; TNM stage ≥III, 41.1 vs 66.2%, p = 0.061). Colorectal cancer patients with liver cirrhosis have poorer OS compared to those without liver cirrhosis; however, the PRF rates are similar. It might be due to the mortality from the liver, and surgical treatment should be actively considered for patients with MELD-Na score <10.

## Introduction

Liver cirrhosis is the final stage of all chronic liver diseases and is considered the main reason for the development of portal hypertension or hepatic encephalopathy [1, 2]. Recently, advances in medicine and surgery, such as liver transplantation to treat cirrhosis, have led to gradual improvements in survival; however, morbidities and mortalities post-abdominal surgery have not substantially decreased [3]. Portal hypertension, which accompanies liver cirrhosis, reduces the systemic arterial blood flow and results in hypotension and hypoxemia, frequently causing liver damage; this can occur easily during surgery [4–6]. Surgery for patients with liver cirrhosis is often restricted to those cases where treatment is not possible using conservative methods. However, surgical treatment must be prioritized if such patients develop colorectal cancer, despite the fact that postoperative morbidity and mortality rates in these patients are increased compared to the general population and remain elevated even after colorectal surgery. Surgery is therefore burdensome for both patients and surgeons [7]. Furthermore, deciding whether to administer chemotherapy to patients with liver cirrhosis is not straightforward, as liver function and systemic conditions must be taken into consideration. Hence, approaches to postoperative treatments in these patients might differ from approaches in patients without liver cirrhosis. Moreover, although the most common site of distant metastasis for colorectal tumors is the liver, reports suggest that liver metastases do not commonly occur in patients with liver cirrhosis [8]. However, studies on this topic are lacking; there have been very few comparisons of the oncologic outcomes of colorectal patients with liver cirrhosis and those without cirrhosis.

In this study, we investigated treatment methods for colorectal cancer, as well as the oncologic and surgical outcomes of colorectal cancer surgery in patients with liver cirrhosis compared to those without liver cirrhosis. Furthermore, we determined prognostic factors for overall survival (OS) and proportion of recurrence free (PRF), particularly those pertaining to patients with liver cirrhosis.

## Materials and methods

### Study population and patient selection

The study included 5,445 patients extracted from a prospectively collected database of patients who underwent surgery for colorectal cancer at our hospital between 2002 and 2010. This study was reviewed and approved by the Institutional Review Board of the Seoul National University Hospital and this study was waived of informed consent. We selected patients whose medical records confirmed that they visited the hospital for complications or symptoms related to liver cirrhosis (i.e., late-stage fibrosis of the liver). Patients with familial adenomatous polyposis or hereditary non-polyposis colorectal cancer were excluded from the study. There were 275 patients enrolled: 55 who had liver cirrhosis before surgery (the LC group) and 220 patients without liver cirrhosis (the non-LC group). The patients underwent 1:4 propensity matching according to sex, age, cancer location, and stage. The body mass indexes (BMIs), Charlson comorbidity scores, and carcinoembryonic antigen (CEA) levels of patients were determined before surgery. To calculate the preoperative model for end-stage liver disease (MELD) score in patients with liver cirrhosis, as well as that of the modified model that includes serum sodium (MELD-Na), we determined the prothrombin time and international normalized ratio (PT/INR), and serum creatinine, serum bilirubin, and serum sodium levels. Specifically, the MELD score is calculated using the patient's serum bilirubin, serum creatinine and PT / INR. It is calculated according to the following formula: *MELD = 3*.*78 × ln [serum bilirubin (mg / dL)] + 11*.*2 × ln [INR] + 9*.*57 × ln [serum creatinine (mg / dL)] + 6*.*43*. For the MELD-Na score, serum sodium is added to the MELD formula. The formula is *MELD Score–serum sodium (mmol/L) - 0*.025 * MELD * [140 –serum sodium (mmol/L)] + 140.

We also investigated the causes of liver cirrhosis as well as the development and treatment of hepatocellular carcinoma (HCC) before and after surgery. Based on the results of previous studies, the cut-off values for the MELD and MELD-Na scores were set at 9 and 10, respectively [9–11].

### Surgery and follow up

In terms of surgical treatment for colorectal cancer, all enrolled patients underwent high ligation, while patients with rectal cancer underwent total mesorectal excision. We also reviewed the results of histopathological examinations, including T stage, number of harvested lymph nodes, number of metastatic lymph nodes, angiolymphatic invasion (ALI), venous invasion (VI), and perineural invasion (PNI). We also recorded the duration of surgery, length of hospital stay, and blood loss, as well as whether intraoperative transfusion had been performed and whether the patient was taken to the intensive care unit after surgery. Additionally, postoperative complications were determined using the Clavien-Dindo classification scale, and major complications were defined as those for which surgical, endoscopic, or radiological intervention was necessary (Clavien-Dindo classification ≥3). Thirty-day mortality was defined as any death during hospitalization within 30 days after surgery. We investigated whether patients received postoperative chemotherapy and whether recurrence occurred (and if so, the sites of recurrence). All patients received follow-up every 3 months for the first 2 years, and then every 6 months for the following 3 years. Patients with liver cirrhosis were followed-up at the Department of Internal Medicine at our hospital.

### Statistical analysis

Statistical analysis was performed using SPSS version 22.0 (IBM Corporation, Armonk, NY, USA). To reduce potential confounding effects and treatment selection bias, we conducted propensity score matching. We selected 4 factors that could affect operative outcomes as follows: age, gender, cancer location and cancer stage. R programming language (version 3.2.5; R Foundation for Statistical Computing, Vienna, Austria), package "MatchIt" and "optmatch" were used for propensity score matching [12, 13]. In this study, four basic conditions for propensity matching were used. We performed propensity matching with factors that could be clearly related to cancer survival among the factors from the multivariate analysis. We did not consider factors that differ in relation to liver cirrhosis. Among the patients with liver cirrhosis, the body mass index, underlying disease, and the Charlson comorbidity index scores were inevitably different. However, there was no difference in the ALI, VI, or PNI, which could affect survival, between the two groups.

The Pearson χ^2^ test or Fisher exact test was used for the comparison of categorical variables between the 2 groups, while the Student t-test was used for the comparison of continuous variables. To adjust for the differences in baseline characteristics between the 2 groups, a propensity score was developed using the logistic regression model. OS was defined as the time between surgery and death, and PRF was defined as the proportion of patients without recurrence, with the recurrence-free survival time defined as the time from surgery until the date of the first observation of tumor recurrence in patients without distant metastasis. The Kaplan-Meier method was used to calculate OS and PRF, and a log-rank test was performed to compare the 2 groups. We also used the log-rank test for univariate analysis of clinicopathological factors; factors found significant on univariate analysis were subjected to multivariate analysis using the Cox proportional hazards regression model. *P*-values <0.05 were considered statistically significant.

## Results

### Clinicopathological characteristics

The LC group consisted of 55 patients and the non-LC group consisted of 220 patients. Although they showed similar baseline characteristics, patients of the non-LC group had a higher mean BMI and a greater number of harvested lymph nodes; however, there was no difference between the 2 groups in terms of patients with CEA levels above 5 ng/mL (Table 1). The laparoscopic rates were 7.3% (4/55) in the LC group and 12.7% (28/220) in the non-LC group, with no statistically significant difference (P = 0.259). None of the patients with colon cancer had stoma. In patients with rectal cancer, 4 out of 15 patients (26.7%) in the LC group and 16 of 60 patients (26.7%) in the non-LC group had stoma. The treatment of all rectal cancer patients in this study was determined by a multidisciplinary team. Two of 15 patients (13.3%) in the LC group and 32 of 60 patients (53.3%) in the non-LC group underwent neoadjuvant concurrent chemoradiotherapy for rectal cancer; the difference was significant (*P* = 0.005). Among the 13 patients in the LC group who did not undergo neoadjuvant chemoradiotherapy, 5 patients were diagnosed with early rectal cancer at the preoperative evaluation and did not undergo chemoradiotherapy, and 6 patients had rectal cancer-related symptoms such as obstruction or bleeding and therefore decided to prioritize surgery. In the remaining 2 patients, the multidisciplinary team decided that surgery was preferred, considering the patients’ liver function and compliance. In the LC group, 7 patients with tumor-node-metastasis (TNM) stage IV disease received surgery for symptoms including obstruction or bleeding due to a colorectal tumor. Causes of the development of liver cirrhosis, Child-Pugh score, MELD score, MELD-Na, and occurrence of HCC are summarized in Table 2. Hepatitis B virus (HBV) was the most common reason for liver cirrhosis. There were 21 patients (38.2%) who developed HCC near the time of the surgery; 10 of them (47.6%) already had HCC before surgery, and 11 (52.4%) developed HCC after surgery. There were 13 patients (61.9%) who did not undergo surgical treatment but received transarterial chemoembolization, radiofrequency ablation, or percutaneous ethanol injection therapy as treatment for HCC. Meanwhile, 2 patients (9.5%) only received surgery, 2 (9.5%) received both treatments, and 4 (19.1%) did not receive any treatment. From the patients in the LC group, 3 patients (5.5%) received liver transplantation (1 before colorectal cancer resection and 2 after resection); 2 liver transplantations were from a living donor and 1 was from a cadaveric donor.

**Table 1:** Demographic data and clinical characteristics of patients with liver cirrhosis and without liver cirrhosis.

		LC (N = 55)	Non-LC (N = 220)	P-value
**Sex**				1.000
	**Male**	39 (70.9%)	156 (70.9%)	
	**Female**	16 (29.1%)	64 (29.1%)	
**Age (years)**				0.896
	**Median (IQR)**	63.0 (59–67)	63.0 (58–68)	
**BMI(kg/m^2^)**		21.9 ± 2.9	23.5 ± 3.1	<0.001
**Tumor location**				1.000
	**Colon**	40 (72.7%)	160 (72.7%)	
	**Rectum**	15 (27.3%)	60 (27.3%)	
**Charlson comorbidity index score**		2.7 ± 1.8	0.5 ± 0.9	<0.001
**Cancer stage**				1.000
	**Stage 0**	1 (1.8%)	4 (1.8%)	
	**Stage I**	12 (21.8%)	48 (21.8%)	
	**Stage II**	12 (21.8%)	48 (21.8%)	
	**Stage III**	23 (41.8%)	92 (41.8%)	
	**Stage IV**	7 (12.7%)	28 (12.7%)	
**Angiolymphatic invasion**				0.178
	**Yes**	27 (49.1%)	86 (39.1%)	
	**No**	28 (50.9%)	145 (65.9%)	
**Venous invasion**				0.315
	**Yes**	6 (10.9%)	36 (16.4%)	
	**No**	49 (89.1%)	184 (83.6%)	
**Perineural invasion**				0.434
	**Yes**	12 (21.8%)	38 (17.3%)	
	**No**	43 (78.2%)	182 (82.7%)	
**Metastatic lymph node**		1.8 ± 2.9	2.4 ± 3.7	0.264
**Harvest lymph node**		16.2 ± 7.9	19.6 ± 11.1	0.012
	**<12**	15 (27.3%)	52 (23.6%)	0.574
	**≥12**	40 (72.7%)	168 (76.4%)	
**Preoperative CEA (ng/ml)**				0.239
	**≤5**	41 (74.5%)	156 (74.6%)	
	**>5**	14 (25.5%)	53 (25.4%)	

BMI indicates body mass index; CEA indicates carcinoembryonic antigen.

**Table 2:** Baseline characteristics of patients with liver cirrhosis.

Etiology and Features of Patients with liver cirrhosis		
**Cause of liver cirrhosis**		
	**Hepatitis B-virus**	35 (63.6%)
	**Alcoholic**	8 (14.5%)
	**Hepatitis C-virus**	7 (12.7%)
	**Idiopathic**	3 (5.5%)
	**Autoimmune hepatitis**	1 (1.8%)
	**Primary biliary cirrhosis**	1 (1.8%)
**Child-Pugh Score**		
	**A**	46 (83.6%)
	**B**	9 (16.4%)
	**C**	0 (0.0%)
**MELD score**		
	**≤ 9**	35 (63.6%)
	**>9**	20 (36.4%)
**MELD-Na score**		
	**≤10**	31 (56.4%)
	>10	24 (43.6%)

### Surgical outcomes and chemotherapy

Comparison of surgical outcomes and complications between the 2 groups showed no difference in surgery duration; however, the LC group had a higher blood loss rate during surgery and underwent more intraoperative transfusions. Length of hospital stay was also longer for the LC group, and postoperative complications occurred in this group more frequently. In terms of postoperative complications, the most common event in the LC group that was not related to liver cirrhosis was wound infection (n = 7), followed by ileus (n = 5), incisional hernia (n = 3), intra-abdominal abscess (n = 1), and parastomal hernia (n = 1). Liver cirrhosis-related complications were uncontrolled ascites (n = 6), portosystemic encephalopathy (n = 1), and severe hypoalbuminemia (n = 1). In contrast, wound infection was the most common complication in the non-LC group (n = 12), followed by small bowel obstruction (n = 6), chyle ascites (n = 2), cerebral vascular accident (n = 1), pneumonia (n = 1) and rectovaginal fistula (n = 1). However, there was no case of anastomotic leakage in either group. There was no 30-day mortality in the non-LC group, while 1 patient (1.8%) in the LC group died because of liver failure after surgery. The ratio of patients who underwent postoperative chemotherapy was significantly lower in the LC group, and oxaliplatin-based chemotherapy was the most common in both groups (Table 3). In the LC group, 4 patients among those with stage II disease (33.3%), 13 among those with stage III disease (56.5%), and 5 among those with stage IV disease (71.4%) received chemotherapy. Of the 16 patients who underwent oxaliplatin-based chemotherapy, treatment was discontinued in 3 because of oxaliplatin-related hepatotoxicity. Among these 3 patients, 2 showed thrombocytopenia accompanied by elevated total bilirubin levels, while 1 showed elevated liver enzymes and increased ascites. Additionally, 13 of 16 patients who underwent oxaliplatin-based chemotherapy (excluding the 3 patients who discontinued chemotherapy) received dose reductions of 20–50%. There was no irinotecan- or 5-fluorouracil-related hepatotoxicity in patients who underwent chemotherapy.

**Table 3:** Compared with surgical outcomes and complications between LC and non-LC group.

		LC (N = 55)	Non-LC (N = 220)	P-value
**Operation time (min)**		148 ± 82	136 ± 83	0.331
**Blood loss (ml)**		241 ± 307	141 ± 145	0.001
**Intraoperative transfusion**				<0.001
	**Yes**	8 (14.5%)	5 (2.3%)	
	**No**	47 (85.5%)	215 (97.7%)	
**Postoperative ICU admission**				0.004
	**Yes**	47 (85.5%)	211 (95.9%)	
	**No**	8 (14.5%)	9 (4.1%)	
**Length of stay (day)**				<0.001
**Postoperative complication**				
	**No complication**	35 (63.6%)	202 (91.8%)	
	**Minor complication**	8 (14.5%)	15 (6.8%)	
	**Major complication**	12 (21.8%)	3 (1.4%)	
**Chemotherapy for patients with TNM stage II, III, IV**				<0.001
	**Yes**	22 (40.0%)	149 (88.6%)	
	**No**	20 (60.0%)	19 (11.4%)	
	**Oxaliplatin-based**	16 (72.7%)	99 (66.4%)	
	**5-FU-based**	4 (18.2%)	42 (28.2%)	
	**Irrinotecan-based**	2 (9.1%)	8 (5.4%)	

ICU indicates intensive care unit

### Proportion of recurrence free and overall survival

The median follow-up period was 45.2 months (interquartile range [IQR]: 28.0–70.2 months) in the LC group and 67.3 months (IQR: 43.1–97.8 months) in the non-LC group. We examined incidences of recurrence and observed no statistical difference between recurrence rates between the 2 groups; however, the sites of recurrence were different. In the LC group, the lungs were the most common recurrence site, while in the non-LC group, the liver was the most common recurrence site (Table 4). Of note, patients with stage IV disease were excluded from the analysis of recurrences.

**Table 4:** A comparison of recurrence between LC and non-LC group except patients with TNM stage IV.

		LC (N = 48)	Non-LC (N = 192)	P-value
**Recurrence**				0.119
	**Yes**	11 (22.9%)	29 (15.1%)	
	**No**	37 (77.1%)	163 (84.9%)	
**Recurrence site**				0.028
	**Liver**	1 (9.1%)	15 (51.7%)	
	**Lung**	8 (72.7%)	5 (13.8%)	
	**Peritoneal seeding**	1 (9.1%)	4 (13.8%)	
	**Distant lymph node**	1 (9.1%)	2 (6.9%)	
	**Anastomosis site**	0 (0.0%)	2 (6.9%)	
	**Bone**	0 (0.0%)	1 (3.4%)	

**Fig 1:**
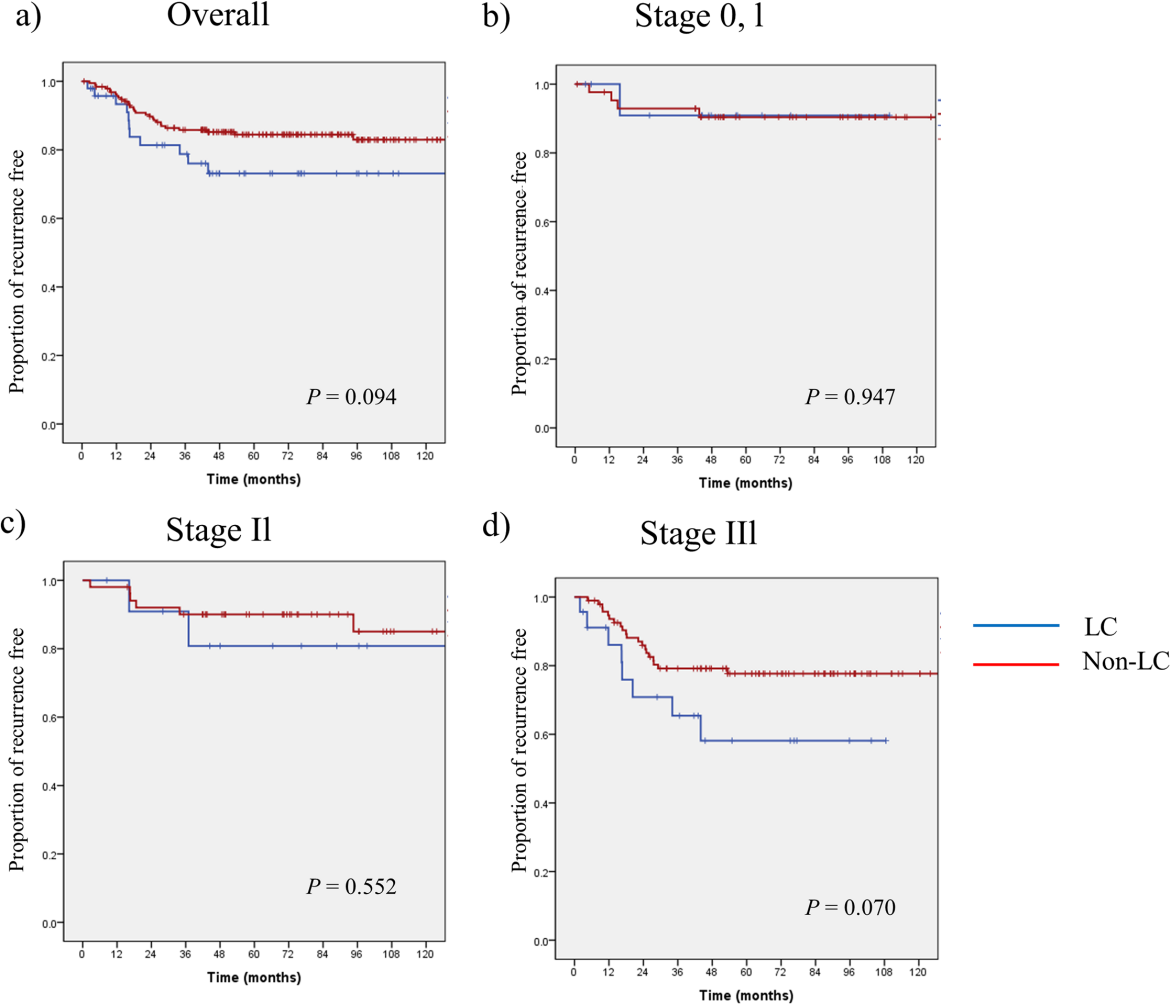
**Kaplan-Meier plots of proportion of recurrence free between LC group and non-LC group a) overall, b) stage 0 and I, c) stage II, and d) stage III**.

Excluding patients with stage IV disease from the 2 groups, the total 5-year PRF was 73.1% in the LC group and 84.5% in the non-LC group, with no significant difference. The PRF rates in patients with stage 0–I disease were 90.9% and 90.4% in the LC and non-LC groups, respectively; again, the difference was not significant. In patients with stage II disease, the PRF rates were 80.8% and 90.0% in the LC and non-LC groups, respectively, while in patients with stage III disease, the PRF rates were 58.1% and 77.7%, respectively. There were no statistical differences between the 2 groups (Fig 1).

**Fig 2:**
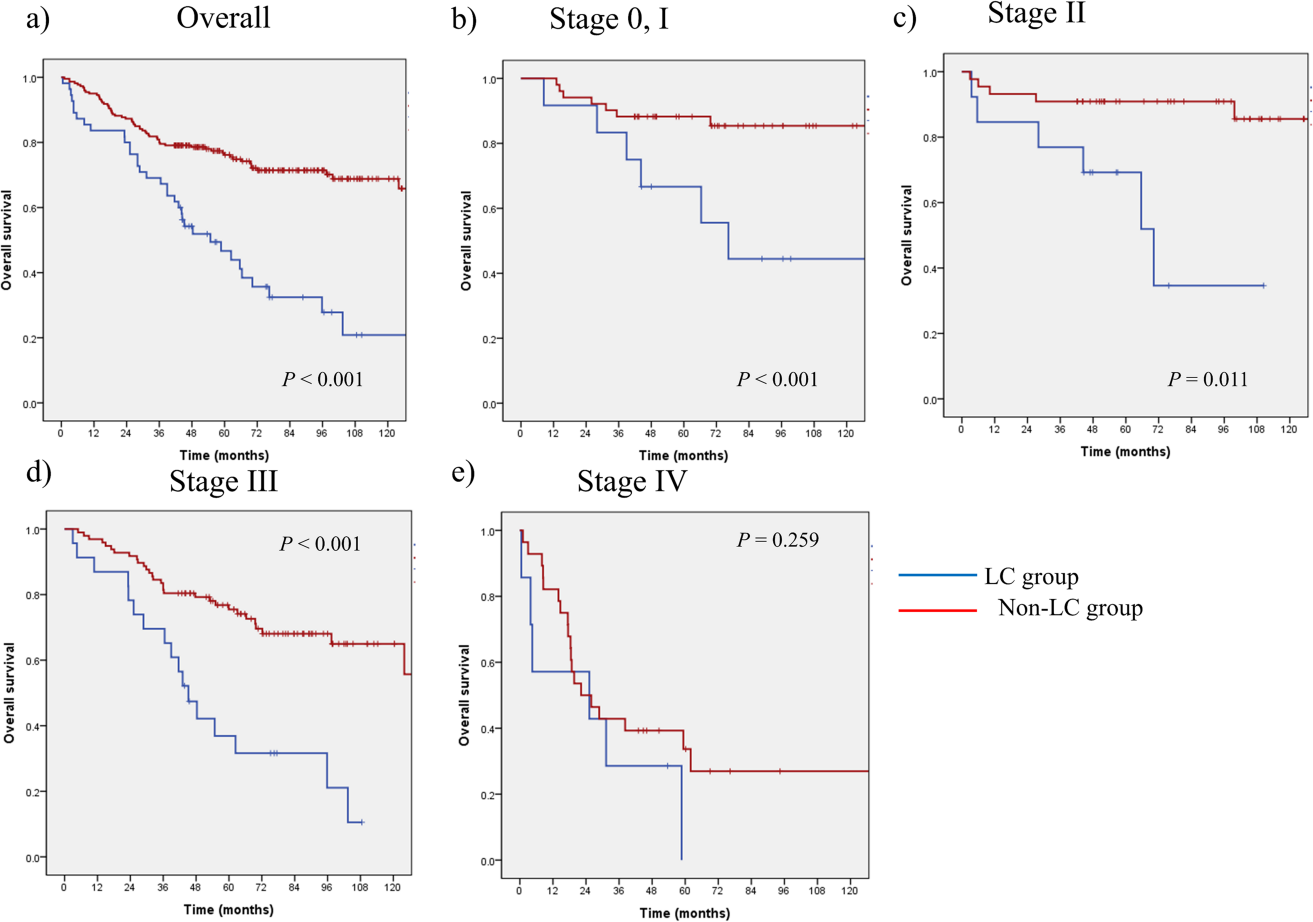
**Kaplan-Meier plots of overall survival between LC group and non-LC group a) overall, b) stage 0 and I, c) stage II, d) stage III and e) stage IV**.

The 5-year OS rate in the LC group (46.7%) was significantly lower than that in the non-LC group (76.2%) (*P* < 0.001). In the LC group, the OS rates for patients with stage 0–I, stage II, stage III, and stage IV cancer were 69.2%, 66.7%, 36.9%, and 0.0%, respectively. The corresponding OS rates for patients in the non-LC group were 90.9%, 88.2%, 75.5%, and 33.7%, respectively. Excluding the OS rate for patients with stage IV disease, the non-LC group showed significantly better OS for all stages (Fig 2). The cause of death in the 35 patients who died in the LC group included liver cirrhosis progression in 24 (68.6%) patients.

### Prognostic factors for survival

On univariate analysis, factors that significantly shortened PRF included TNM stage ≥III disease; location of the tumor in the rectum; undergoing intraoperative transfusion; presence of ALI, VI, and/or PNI; occurrence of postoperative complications; and a preoperative CEA level >5 ng/mL. Univariate analysis also revealed that these same variables, with the exception of tumor location in the rectum, significantly shortened OS rates. On univariate analysis performed only in the LC group, TNM stage ≥III disease, intraoperative transfusion, presence of VI, a preoperative CEA level >5 ng/mL, and a MELD-Na score >10 significantly shortened OS (Table 5).

**Table 5:** Prognostic factors of 5-year survival by univariate analysis.

		N = 240	Proportion of recurrence free (%)	*P*	N = 275	Overall survival (%)	*P*	N = 55	Overall survival for patients with LC(%)	*P*
**Group**				0.114			<0.001			
	**LC**	48	73.1		55	46.7		-	-	-
	**Non-LC**	192	84.5		220	77.6				
**Sex**				0.709			0.395			0.946
	**Male**	174	82.4		198	72.6		39	41.6	
	**Female**	66	80.5		77	64.2		16	44.6	
**Age**				0.594			0.464			0.446
	**≤ 65**	149	82.3		180	68.6		36	40.5	
	**>65**	91	81.2		95	73.8		19	46.9	
**TNM stage**				0.006			<0.001			0.018
	**0, I and II**	120	89.4		125	84.8		25	62.7	
	**III and IV**	120	74.3		150	59.3		30	28.2	
**Cancer location**				0.001			0.395			0.083
	**Colon**	167	87.0		200	69.3		40	45.8	
	**Rectum**	73	70.3		75	72.8		15	44.4	
**Intraoperative transfusion**				0.001			<0.001			0.004
	**No**	229	83.3		262	73.4		47	48.6	
	**Yes**	11	30.5		13	7.7		8	12.5	
**Angiolymphatic invasion**				0.001			<0.001			0.940
	**No**	151	88.6		162	81.5		28	44.2	
	**Yes**	89	70.7		113	55.3		27	42.1	
**Venous invasion**				0.001			<0.001			0.003
	**No**	211	84.9		233	75.3		49	49.2	
	**Yes**	29	58.5		42	42.9		6	0.0	
**Perineural invasion**				<0.001			<0.001			0.082
	**No**	206	86.4		225	76.4		43	47.9	
	**Yes**	34	51.8		50	42.9		12	24.3	
**Complication**				0.050			<0.001			0.142
	**No**	207	83.7		236	74.6		35	46.8	
	**Yes**	33	69.1		39	43.6		20	36.0	
**Chemotherapy (≥ stage II)**		n = 182		0.816	n = 210		0.085	n = 44		0.025
	**No**	34	77.2		39	55.3		20	29.5	
	**Yes**	148	79.9		171	68.8		22	45.8	
**Preoperative CEA (ng/ml)**				0.002			<0.001			0.025
	**≤ 5**	184	86.0		197	77.1		41	50.9	
	**> 5**	46	66.0		67	53.1		14	21.4	
**Hepatocellular carcinoma**		-	-	-	-	-	-			0.140
	**No**							34	51.5	
	**Yes**							21	27.4	
**MELD scores**		-	-	-	-	-	-			0.070
	**≤ 9**							35	46.6	
	**> 9**							20	34.3	
**MELD-Na scores**		-	-	-	-	-	-			0.003
	**≤ 10**							31	54.6	
	**> 10**							24	27.5	

CEA indicates carcinoembryonic antigen; LC, liver cirrhosis; MELD, model for end-stage liver disease; MELD-Na, model for end-stage liver disease with the addition of the serum sodium concentration.

Multivariate analysis revealed that factors that significantly reduced PRF included the presence of rectal tumors as well VI and/or PNI. Moreover, OS was significantly shorter in patients of the LC group as well as those who underwent intraoperative transfusion, exhibited VI and/or PNI, or expressed preoperative CEA level >5 ng/mL. Multivariate analysis performed only on the LC group showed that OS was significantly shorter for patients with TNM stage ≥III disease, those exhibiting VI, and those with a MELD-Na score >10 (Table 6).

**Table 6:** Prognostic factors of 5-year survival by multivariate analysis.

		Proportion of recurrence free HR (95% CI)	P*	Overall survival HR (95% CI)	P*	Overall survival for patients with LC HR (95% CI)	P*
**Group**		-	-		<0.001	-	-
	**LC**			1			
	**Non-LC**			2.54 (1.51–4.27)			
**TNM stage**			0.089		0.134		0.046
	**0, I and II**	1		1		1	
	**III and IV**	1.06 (0.47–2.35)		1.53 (0.88–2.66)		2.45 (1.02–5.89)	
**Cancer location**			0.001	-	-	-	-
	**Colon**	1					
	**Rectum**	3.05 (1.54–6.03)					
**Intraoperative transfusion**			0.766		0.030		0.451
	**No**	1		1		1	
	**Yes**	1.25 (0.29–5.40)		2.41 (1.09–5.34)		1.55 (0.49–4.87)	
**Angiolymphatic invasion**			0.051		0.486	-	-
	**No**	1		1			
	**Yes**	2.15 (0.99–4.66)		1.20 (0.73–1.97)			
**Venous invasion**			0.030		<0.001		0.009
	**No**	1		1		1	
	**Yes**	2.40 (1.09–5.30)		2.41 (1.43–4.06)		4.30 (1.44–12.86)	
**Perineural invasion**			0.001		0.013	-	-
	**No**	1		1			
	**Yes**	3.41 (1.61–7.23)		1.83 (1.14–2.94)			
**Preoperative CEA (ng/ml)**			0.190		0.044		0.916
	**No**	1		1		1	
	**Yes**	1.45 (0.83–2.52)		1.60 (1.01–2.52)		1.05 (0.39–2.82)	
**Complication**			0.260		0.303	-	-
	**≤ 5**	1		1			
	**> 5**	1.82 (0.64–5.19)		1.38 (0.75–2.56)			
**MELD-Na scores**		-	-	-	-		0.041
	**≤ 10**					1	
	**> 10**					2.56 (1.04–6.32)	

* Cox proportional hazards regression model. CEA indicates carcinoembryonic antigen; CI, confidence interval; HR, hazard ratio; LC, liver cirrhosis; MELD-Na, model for end-stage liver disease with the addition of the serum sodium concentration.

**Fig 3:**
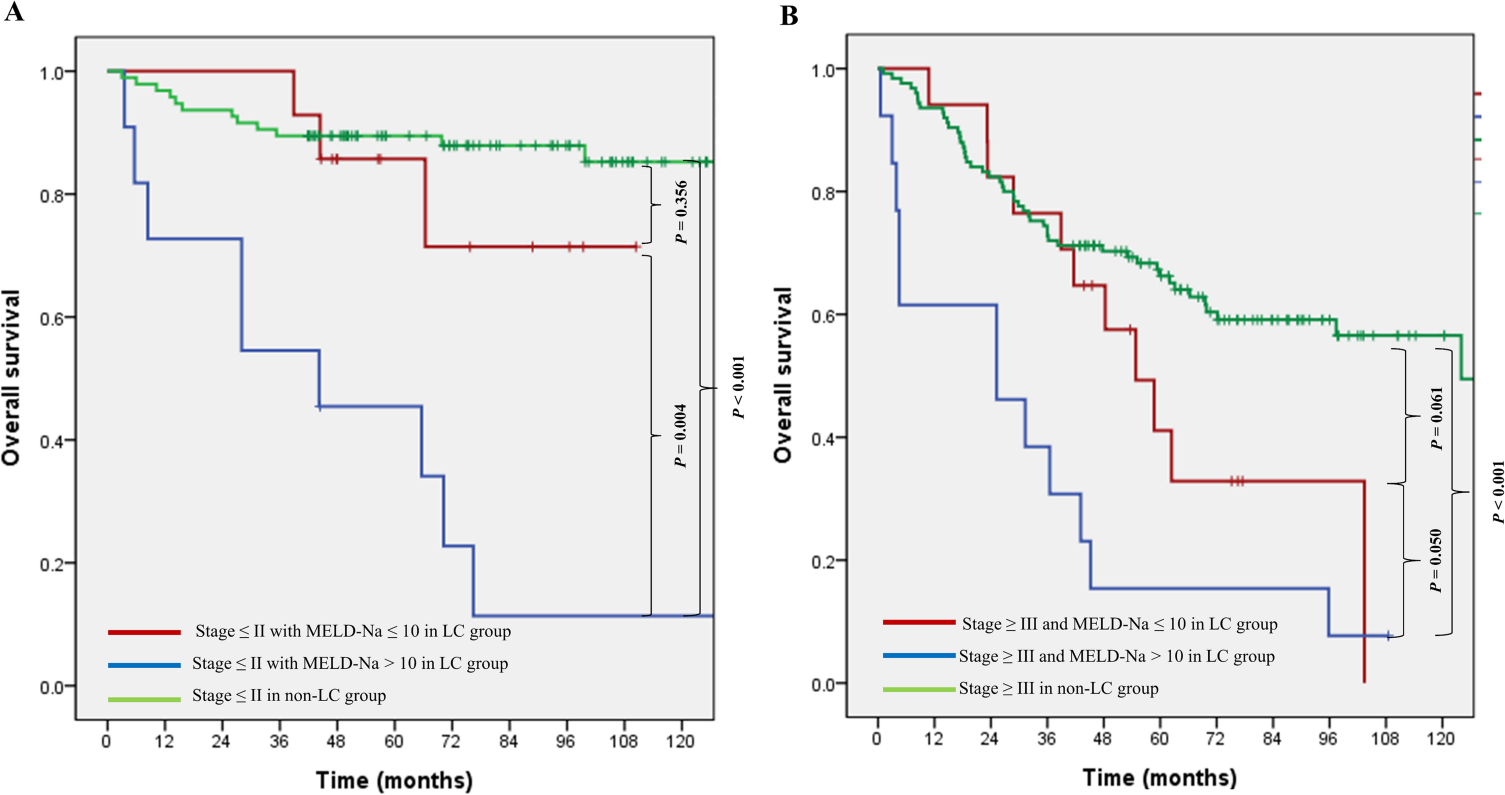
Kaplan-Meier plots of overall survival in LC group according to cancer stage and MELD-Na score.

We investigated survival by classifying patients based on the threshold criteria of stage II disease and a MELD-Na score of 10. The 5-year OS rate of non-LC group patients with stage ≤II disease was 89.5%, and those of LC group patients with stage ≤II disease and MELD-Na score ≤10, LC group patients with stage ≤II disease and MELD-Na score >10 were 85.7%, 45.5%, respectively. The 5-year OS rate of non-LC group patients with stage ≥III disease were 66.2%, and those of LC group patients with stage ≥III disease and MELD-Na score ≤10, LC group patients with stage ≥III disease and MELD-Na score >10 were 41.1%, 7.7%, respectively. Although the OS of LC group with MELD-NA scores >10 had the worse result both stage ≤II and ≥III disease, there were no differences between LC group with MELD-Na score ≤10 and non-LC group (p = 0.356, 0.061) (Fig 3).

## Discussion

The present study revealed that patients with liver cirrhosis have a higher rate of postoperative complications. Postoperative 5-year OS was lower in the LC group than in the non-LC group. However, there was no difference in 5-year PRF rates between the groups. For OS, the hazard ratio for the LC group was 2.5-fold worse than that for the non-LC group. Furthermore, we found that the prognosis for colorectal cancer patients with LC was poorer when advanced stage III or VI tumor was present, and the MELD-Na score was >10.

Liver cirrhosis was the most important prognostic factor for OS in this study, but PRF was not different compared to that in patients in the non-LC group. The survival rate could be affected significantly by the poor liver function and mortality from liver cirrhosis. Previous studies that examined survival rates among patients with liver cirrhosis found that 1-year mortality was 1–3.4% in patients with compensated cirrhosis and 20–57% in those with decompensated cirrhosis [14], suggesting that patients with liver cirrhosis show varying survival rates according to liver function. Other studies have used tools such as the preoperative Child-Turcotte-Pugh (CTP) and MELD scores to report on the absolute necessity of considering postoperative morbidity and mortality [15]. The CTP score has generally been used to assess the severity of liver cirrhosis and predict prognosis. However, its disadvantage is that the scoring of ascites and hepatic encephalopathy, both of which are CTP variables, can vary among physicians [16]. The MELD score was developed to predict the short-term mortality of patients who received transjugular intrahepatic portosystemic shunts, and has since been used to predict the mortality of liver diseases in various studies. The score is calculated using serum creatinine, serum bilirubin, and PT/INR. As studies demonstrate that serum sodium is an independent factor for the prognosis of patients with liver cirrhosis, the MELD-Na score, which supplements the MELD score, has also been used in recent years [17]. The present study involved retrospective analyses; survival was analyzed using MELD and MELD-Na scores because it was difficult to investigate CTP scores to indicate liver function. The MELD scores were divided into those below 9 points, which were associated with a 5-year OS rate of 46.6%, and those above 9 points, which were associated with a 5-year OS rate of 34.3%, although the difference was not significant. However, after examining the 5-year OS by setting 10 as a threshold value for the MELD-Na score, we found the rates to be 54.6% and 27.5% below and above this threshold, respectively, showing poorer survival as the MELD-Na score increased. Furthermore, when the MELD-Na score was <10, OS was not different compared with that of the non-LC group. Previous studies also demonstrated that the MELD-Na score was a better prognostic factor for patients with liver cirrhosis, compared to the MELD score [18, 19]. The present study classified survival according to the TNM stage and MELD-Na score; both were determined to be significant factors on multivariate analysis. When the MELD-Na score is <10, surgical treatment should be actively considered for colorectal cancer patients with liver cirrhosis. When the MELD-Na score is >10, treatment should be undertaken to improve the MELD-Na score to <10 to achieve better survival outcomes.

Although there was no statistical significance, the PRF of the patients with stage III disease was lower in the LC group, possibly due to the lower rate of postoperative chemotherapy in this group. When analyzed according to TNM stage, we found that, among patients with TNM stage III tumors, the recurrence rate tended to be higher in the LC group (22.9%) than in the non-LC group (15.1%); however, the difference was not statistically significant (p = 0.119). The reason for this finding is likely the small sample size of patients with TNM Stage III tumors in the LC group. Further studies with a large number of patients are needed to verify the safety and efficacy of chemotherapy in these patents. In addition, there are little data on chemotherapy for liver cirrhosis patients because such patients are generally excluded from analysis in randomized controlled trials [20, 21]. A previous study recommended taking into account liver-related and tumor-related prognoses when considering treatment for patients with life expectancies ≥3 months [22]. Currently, there is a lack of consensus or guidelines regarding chemotherapy for colorectal cancer patients with cirrhosis. When administrating chemotherapy to such patients, liver function must be taken into consideration, and the presence of portal hypertension should be examined. Anticancer drugs must be selected with caution [23], and hepatotoxicity must be considered when administrating chemotherapy to these patients. In particular, the anticancer drug oxaliplatin is known to cause sinusoidal obstruction syndrome [24]. A previous study reported that oxaliplatin-based chemotherapy failed to significantly reduce cancer-specific mortality in patients with portal hypertension, irrespective of liver cirrhosis occurrence, and increased the overall morbidity and mortality [25]. Of the 22 patients in our study who received chemotherapy, 16 (72.7%) received oxaliplatin-based chemotherapy. Of these, 3 patients (18.8%) exhibited hepatotoxicity due to oxaliplatin; chemotherapy was discontinued for all. Moreover, oxaliplatin dose reduction was required for 13 patients in the current study. The 5-year OS of patients in the LC group who received chemotherapy was higher but not significantly so; hence, chemotherapy was not a significant prognosis factor for OS. While our results did not show significant oncologic outcomes associated with the administration of chemotherapy in colorectal cancer patients with cirrhosis, it would be difficult to generalize these results owing to the small sample size. More studies on this subject are warranted in the future.

Furthermore, although liver cirrhosis is not a contraindication to concurrent chemoradiotherapy, rectal cancer patients in the LC group had a lower rate of concurrent chemoradiotherapy in this study. Because it is important to consider the various complications that may arise when performing surgery after neoadjuvant chemotherapy, we considered that if complications developed postoperatively, the patients with LC were unlikely to recover. Moreover, the liver cirrhosis can worsen during the period of neoadjuvant treatment, thereby increasing the risk of complications. Accordingly, only 8 patients (14.5%) in the LC group were treated with concurrent chemoradiotherapy. Due to this small sample size, further studies on the safety and efficacy of concurrent chemoradiotherapy in LC patients are needed. It should also be noted that there was no case of anastomotic leak among the patients in this study. It was confirmed in the COREAN trial (1.2%) [26] that Asians generally have a lower rate of leakage after colorectal cancer surgery than Westerners (11–12%) [27]. The reason for this study is that the patients who are more likely to have leakage are selected for stoma formation and the proportion of rectal cancer is lower than that of Westerners. We thought that the lower rate of adjuvant chemotherapy in LC patients might result from the lower compliance for chemotherapy due to their poor general physical conditions. In this study, the proportion of patients with rectal cancer was designed to be the same between the two groups. We evaluated that morbidity was not heterogenous by including rectal cancer patients. The incidence of complications in two groups of rectal cancer patients was higher in the LC group (n = 9, 60%) than in the non-LC group (n = 7, 11.7%). But, the complications related to rectal cancer surgery such as intraabdominal abscess or pelvic abscess were not different between LC group (n = 1, 6.7%) and non-LC group (n = 1, 1.7%) (p = 0.362). According to the results of this study, the LC group with rectal cancer also seemed to be more affected by the LC related factors such as liver function than the cancer related factor in the case of overall survival. Thus, we considered that the neoadjuvant CCRT for rectal cancer did not affect the difference of survival outcomes between the two groups.

Metastases to the liver are reported to be infrequent in patients with liver cirrhosis [28]. Previous studies also showed that patients with colorectal cancer rarely exhibit metastases to injured livers, especially those that are cirrhotic [8, 29–32]. We considered several hypotheses that may explain this phenomenon. One is that tumor cells fail to enter the liver because of reduced blood flow, owing to portal hypertension [33]. Another hypothesis is that a physical barrier is formed after a process that is referred to as ‘capillarization’, leading to the formation of a basement membrane owing to changes in the fenestrated endothelium [8]. A third hypothesis, based on animal studies, posits that a cirrhotic liver per se is not suitable for metastatic cells to take hold. A study showed that Kupffer cells in cirrhotic livers sensitize metastatic colon cancer cells to FasR-mediated apoptosis by up-regulating their expression of Fas receptors, enabling their elimination by tumor-infiltrating lymphocytes. This demonstrates the existence of an immunologic impediment to hepatic colonization by colon cancer cells [34]. Lastly, a fourth hypothesis proposes the existence of a high concentration of metalloproteinase inhibitors in cirrhotic livers, compared to normal livers, producing resistance to metastasis [35]. However, none of the aforementioned hypotheses have been confirmed yet. According to our data, recurrence in patients with liver cirrhosis more often involved metastasis to the lungs compared to recurrence in patients without cirrhosis. For this reason, colorectal cancer patients with liver cirrhosis should undergo imaging tests, including chest computed tomography.

We found that intraoperative transfusion was a prognostic factor for OS. According to previous studies, perioperative transfusion can lead to complications such as pneumonia, surgical site infection, and sepsis; perioperative transfusion has also been identified as an independent factor associated with mortality [36, 37]. When performed for colorectal cancer patients in particular, perioperative allogeneic red blood cell transfusion can have a negative impact on oncologic outcomes in terms of disease recurrence and OS [38–40]. While it is assumed that the mechanism is associated with transfusion-related immunomodulation, this has yet to be verified [41]. Moreover, coagulation factors are not synthesized in the livers of patients with cirrhosis. Because bleeding tendency is high in patients with cirrhosis compared to normal individuals, the risk of bleeding during surgery is also higher [42]. Portal hypertension is responsible for retroperitoneal varices and fibrosis that are the main reasons for bleeding during colorectal surgery; massive intraoperative variceal bleeding causes systemic hypotension, postoperative morbidity, and postoperative mortality. However, total mesocolon and mesorectum excisions are critical for favorable oncologic results; therefore, these procedures cannot be avoided. As our study showed, there was significantly more blood loss during surgery in the LC group; more patients in this group also underwent transfusion during surgery compared to patients in the non-LC group. Furthermore, this unintentional bleeding interfered with the surgeon’s line of sight, possibly influencing the harvesting of lymph nodes, as demonstrated in our study.

The cancer stage was not a significant prognostic factor in the multivariate analysis performed in all patients included in this study. However, the univariate and multivariate analyses of OS in patients in the non-LC group showed that TNM stage, chemotherapy, ALI, VI, PNI, and elevated CEA were significant prognostic factors. These results indicate that the prognostic factors of patients in the LC group after surgery may be different from those in non-LC patients. Likewise, univariate and multivariate analyses of prognostic factors for the PRF in patients in the non-LC group revealed significant associations. As we showed, the MELD-Na score was the most important prognostic factor in LC patients. As most of the patients died with LC progression without the recurrence of the tumor, the recurrence and survival would not be different according to stage in LC group in this study. Likewise, univariate and multivariate analysis of prognostic factors for PRF in patients with non-LC group also shows significant results.

The limitations of our study were the small sample size of patients with liver cirrhosis and the fact that all patients were Asian. Liver cirrhosis due to HBV infection is common in Eastern countries, and approximately 5% of the adult population in South Korea is estimated to be infected with HBV; this rate is high compared to that observed in other countries [43]. Because 15–40% of these patients are at risk of developing liver cirrhosis and HCC, they are regularly screened for liver cancer [44, 45]. Hepatitis C virus infection and alcoholism are common causes of liver cirrhosis in Western countries; therefore, further studies on Western populations are required. The incidence of colon cancer has been increasing among liver cirrhosis patients, but there are only a few studies on patients with colorectal cancer and liver cirrhosis. In particular, studies of prognostic factors and survival rate comparisons using case-matched patients without liver cirrhosis are lacking; hence, the findings of the current study are important. Another limitation was the non-uniformity of treatment regimens for the patients with liver cirrhosis included in the study.

In conclusion, although the postoperative complications can be higher and the OS can be poorer in the patients with liver cirrhosis, we observed no difference in PRF compared to those without liver cirrhosis. Surgical treatment for colorectal cancer should be actively considered in patients with MELD-Na scores <10. Improving liver function could be the most important factor for achieving better OS in patients who have colorectal cancer with liver cirrhosis.
